# The cognitive thalamus

**DOI:** 10.3389/fnsys.2015.00039

**Published:** 2015-03-17

**Authors:** Yuri B. Saalmann, Sabine Kastner

**Affiliations:** ^1^Department of Psychology, University of Wisconsin – MadisonMadison, WI, USA; ^2^Department of Psychology and Princeton Neuroscience Institute, Princeton UniversityPrinceton, NJ, USA

**Keywords:** mediodorsal thalamus, anterior thalamus, intralaminar thalamus, thalamocortical interactions, pulvinar

The thalamus, once viewed as passively relaying sensory information to the cerebral cortex, is becoming increasingly acknowledged as actively regulating the information transmitted to cortical areas. There are a number of reasons for this change. First, evidence suggests that first-order thalamic areas, like the lateral geniculate nucleus, ventral division of the medial geniculate nucleus, and the ventral posterior nuclei, can modulate neural processing along the sensory pathways to the cortex according to behavioral context (O'Connor et al., 2002; McAlonan et al., 2008). Second, much of the thalamus receives relatively little input from the sensory periphery, instead receiving its major driving input from the cortex. This higher-order thalamus forms pathways between cortical areas, which can strongly influence cortical activity (Theyel et al., 2010; Purushothaman et al., 2012; Saalmann et al., 2012). Third, lesions to higher-order thalamic areas, such as the pulvinar and mediodorsal nucleus, can produce severe attention and memory deficits (Saalmann and Kastner, 2011; Baxter, 2013; Bradfield et al., 2013; Jankowski et al., 2013; Mitchell and Chakraborty, 2013), suggesting an important role for the thalamus in cognition.

In this Research Topic, we bring together neuroscientists who study different parts of the thalamus, particularly the higher-order thalamic nuclei, to highlight thalamic contributions to learning (Bradfield et al., 2013; Habib et al., 2013), memory processes (Baxter, 2013; Funahashi, 2013; Jankowski et al., 2013; Mitchell and Chakraborty, 2013; Saalmann, 2014), set-shifting (Bradfield et al., 2013; Minamimoto et al., 2014; Saalmann, 2014), language (Klostermann et al., 2013), as well as movement monitoring and control (Ostendorf et al., 2013; Prevosto and Sommer, 2013; Minamimoto et al., 2014). These studies incorporate a range of methods, from molecular to systems-level approaches, and connect rodent, non-human primate and human data, for a better understanding of human cognition.

The first three articles focus on movement monitoring and motor control. Based on lesion data from clinical subjects, Ostendorf et al. (2013) show that the central thalamus makes an important contribution to predicting the perceptual consequences of eye movements. Focusing on cerebello-cortical pathways incorporating the central and ventral lateral thalamus, Prevosto and Sommer (2013) review evidence for thalamic modulation of movement processing based on cognitive requirements. Encompassing a number of thalamic nuclei, including the pulvinar, mediodorsal and ventral intermediate nuclei, Klostermann et al. (2013) discuss contributions of the thalamus and basal ganglia to language perception and production.

The Research Topic continues on the theme of behavioral flexibility. Minamimoto et al. (2014) show that that the macaque centromedian nucleus, in the intralaminar thalamus, plays a role in counteracting behavioral biases, which contributes to flexible behavior via interactions with the basal ganglia. Bradfield et al. (2013) review evidence from rodent studies that another intralaminar thalamic nucleus, the parafascicular thalamus, also contributes to behavioral flexibility, whereas the mediodorsal thalamic nucleus plays a key role in acquiring goal-directed behavior.

Next, the focus shifts to memory processes. Jankowski et al. (2013) review contributions of the anterior thalamus, and its interactions with the hippocampus and cortex, to memory processing and spatial navigation in rodents. This includes evidence for oscillatory activity at theta frequencies in the anterior thalamus. Habib et al. (2013) investigate memory processes in the auditory thalamus, showing differential molecular events underlying safety learning and fear conditioning.

Finally, there are four reviews highlighting different functions of the large mediodorsal thalamic nucleus and its interactions with the prefrontal cortex in primates. Mitchell and Chakraborty (2013) discuss the effects of lesions of the mediodorsal thalamus, supporting its role in memory and other cognitive processes. Baxter (2013) argues that the mediodorsal thalamus regulates plasticity within prefrontal cortex as well as the flexibility of prefrontal-dependent operations. Funahashi (2013) reviews contributions of the mediodorsal thalamus to spatial working memory, including how interaction between the thalamus and prefrontal cortex can enable sensory-to-motor transformations of maintained information. To conclude, Saalmann (2014) proposes that the mediodorsal thalamus regulates synchrony between neurons in prefrontal cortex and, consequently, their information exchange according to cognitive control demands.

This Research Topic highlights the key contributions of the thalamus to neural processing in cortico-cortical, hippocampo-cortical, cortico-striatal and cerebello-cortical pathways. Although the underlying mechanisms of thalamic influence on these pathways remain to be clarified, there is growing evidence that the thalamus plays a key role in dynamically routing information across the brain (Saalmann et al., 2012; Xu and Sudhof, 2013). Such a role may involve flexibly synchronizing ensembles of neurons, thereby configuring brain networks for the current behavioral context. Taken together, the articles in this Research Topic show that thalamic interactions with cortical and subcortical areas are integral to behavioral flexibility, memory processes and cognition in general.

## Conflict of interest statement

The authors declare that the research was conducted in the absence of any commercial or financial relationships that could be construed as a potential conflict of interest.
